# The nanos^d^ integral gene drive enables population modification of the malaria vector *Anopheles gambiae*

**DOI:** 10.1093/g3journal/jkaf246

**Published:** 2025-10-29

**Authors:** Pei-Shi Yen, Sebald A N R Verkuijl, Paolo Capriotti, Giuseppe Del Corsano, Calvin Kim Guan Yee, Astrid Hoermann, Maria Grazia Inghilterra, Irati Aramburu-Gonzalez, Moeez A Khan, Dina Vlachou, George K Christophides, Nikolai Windbichler

**Affiliations:** Department of Life Sciences, Imperial College London, London SW7 2AZ, England, United Kingdom; Department of Life Sciences, Imperial College London, London SW7 2AZ, England, United Kingdom; Department of Life Sciences, Imperial College London, London SW7 2AZ, England, United Kingdom; Department of Life Sciences, Imperial College London, London SW7 2AZ, England, United Kingdom; Department of Life Sciences, Imperial College London, London SW7 2AZ, England, United Kingdom; Department of Life Sciences, Imperial College London, London SW7 2AZ, England, United Kingdom; Department of Life Sciences, Imperial College London, London SW7 2AZ, England, United Kingdom; Department of Life Sciences, Imperial College London, London SW7 2AZ, England, United Kingdom; Department of Life Sciences, Imperial College London, London SW7 2AZ, England, United Kingdom; Department of Life Sciences, Imperial College London, London SW7 2AZ, England, United Kingdom; Department of Life Sciences, Imperial College London, London SW7 2AZ, England, United Kingdom; Department of Life Sciences, Imperial College London, London SW7 2AZ, England, United Kingdom

**Keywords:** genetics, synthetic biology, malaria, gene drive, engineering biology, genetic control

## Abstract

The modification of mosquito populations at scale through CRISPR-Cas9-mediated homing gene drives is a promising route for malaria vector control. Integral gene drives (IGDs) are designed to utilize the regulatory sequences of endogenous genes to reduce the size of the modification required for nuclease and effector expression. In this study, we describe the creation and characterization of the nanos^d^ integral gene drive, which targets and is inserted into the *nanos* gene of the malaria vector *Anopheles gambiae*, and show that it achieves high rates of gene drive (98.4% in females, 99.5% in males). We find that homozygous nanos^d^ females but not males show impaired fecundity and exhibit variable degrees of ovary underdevelopment. Transcriptomic analysis of ovaries points to decreased transcript levels of the *nanos* gene when harboring Cas9 and changes to other fertility-related genes. As a minimal genetic modification, nanos^d^ does not induce widespread transcriptomic perturbations that would affect vector competence, and we show that its susceptibility to *Plasmodium* spp. and O’nyong nyong virus infection remains similar to wild-type mosquitoes. Importantly, we find that nanos^d^ propagates efficiently in caged mosquito populations and is maintained as a source of Cas9 after the emergence of drive-resistant alleles, whilst also mobilizing a nonautonomous antiparasitic effector modification. The nanos^d^ gene drive shows promise as a genetic tool for malaria vector control via population modification, and we outline steps towards its further optimization.

## Introduction

Malaria is the deadliest infectious disease transmitted by mosquitoes. In 2023, an estimated 263 million malaria infections occurred, leading to 597,000 deaths (Venkatesan 2024). This follows a trend of increased infection and death incidences in recent years, driven by, among others, the spread of drug, diagnostic, and insecticide resistance. Additionally, climate change is altering vector distribution and influencing the malaria transmission cycle (Afrane et al. 2012), while the lingering healthcare disruptions from the COVID-19 pandemic have further weakened prevention and treatment efforts (Weiss et al. 2021; Venkatesan 2024 ). This underpins the need for alternative tools and strategies for malaria control, particularly methods that target mosquito vectors and reduce transmission. Among the proposed strategies for vector control, homing gene drive systems are considered one of the most promising tools to achieve malaria eradication (Feachem et al. 2019). Gene drives are genetic elements engineered to bias their own inheritance, facilitating the spread of specific traits within a target population. Gene drive systems can allow traits of interest to increase in frequency across generations, even in the absence of a fitness advantage. The advancement of the CRISPR-Cas9 endonuclease platform has significantly accelerated the development of gene drive technologies for public health and pest control applications (Legros et al. 2021; Naidoo and Oliver 2025). In a homing gene drive system, the CRISPR-Cas9 nuclease induces a DNA double-strand break at a specific locus. The cellular repair machinery then initiates repair from the homologous sequences on the other chromosome that contains the gene drive components. This “homing” mechanism converts the target sequence into a gene drive carrying allele in the germline, thus increasing drive inheritance.

Several homing drives have demonstrated high levels of biased inheritance in *Anopheles* mosquitoes (Gantz et al. 2015; Carballar-Lejarazú et al. 2020; Hammond et al. 2021; Carballar-Lejarazú et al. 2023; Green et al. 2023; Verkuijl et al. 2025). Homing drives typically consist of a Cas9 coding sequence driven by germline-specific regulatory elements, and a gRNA expressed under the control of an RNA Polymerase III promoter. The fitness costs, homing efficiency, and resistance rate of the gene drive are strongly influenced by the nuclease activity window. Activity in nongermline tissues, through “leaky” expression or parental nuclease deposition into the zygote, can be detrimental if the Cas9-gRNA-mediated DNA break cannot be repaired via homology-directed repair (HDR). When they target and disrupt essential mosquito genes, gene drives are designed to ensure that the nuclease activity window (germline) does not overlap with that of the target gene (soma). This ensures that DNA repair occurs in the germline, where HDR is favored and avoids somatic nuclease activity that could lead to loss-of-function phenotypes in heterozygote drive carriers (Fuchs et al. 2021; Hammond et al. 2021), although somatic expression may be more tolerable with drives incorporating effective rescue elements. It is challenging to achieve germline-restricted expression, and gene drive optimization efforts generally focus on testing a range of putative regulatory elements from germline-specific genes to drive Cas9 expression (Hammond et al. 2017; Champer et al. 2020; Anderson et al. 2023). Optimization has relied on the laborious testing of many candidate regulatory sequences (Kyrou et al. 2018; Carballar-Lejarazú et al. 2020; Anderson et al. 2023; Du et al. 2024), yet suboptimal performance can still result from the failure to capture all relevant regulatory elements required for a gene's faithful expression pattern (often only a promoter fragment, 5′ UTR, and 3′ UTR are used), or the interference from cis-regulatory elements at the transgene's integration site (*e.g.* enhancers; Fuchs et al. 2021; Hammond et al. 2021).

In contrast to conventional homing gene drives, integral gene drives (IGDs) seek to utilize the full regulatory context of an endogenous germline gene locus, making it more likely that the intended expression pattern is achieved (Nash et al. 2018). To achieve this, they are inserted in-frame within, and maintaining the function of, an endogenous germline gene. For integral drive elements, there are no intended recessive fitness costs of the insertion, and as such activity of the nuclease can overlap with that of the target gene in the germline. IGDs benefit from a reduced genetic footprint and from the fact that frameshift mutations or disruptions of regulatory elements would impair host gene function and would be subsequently selected against. IGDs have been demonstrated in *Drosophila melanogaster*, *Mus musculus*, *Anopheles stephensi*, and *Anopheles gambiae*, and while allowing for efficient homing rate, they can also incur severe unintended fitness costs, in particular from impaired host gene expression (Weitzel et al. 2021; Ellis et al. 2022; Nash et al. 2022; Gonzalez et al. 2025). For germline host genes the likely result is reduced mosquito fertility which in turn hampers gene drive propagation in the target population. Additional efforts are required to identify suitable host gene loci and gene drive cassette designs that allow for optimal IGD performance.

The *nanos* gene is part of a highly conserved gene family found in both vertebrates and invertebrates (Curtis et al. 1995; Tsuda et al. 2003). In *A. gambiae*, *nanos* expression is restricted to embryos (Calvo et al. 2005; Terradas et al. 2021) and the germline of both sexes, where it encodes a zinc-finger RNA-binding domain involved in regulating the translation of mRNAs critical for polarity and germ cell development in embryos (Rongo and Lehmann 1996; De Keuckelaere et al. 2018). Regulatory sequences (including promoter sequences and UTRs) of the *nanos* gene have been utilized in gene drives and other genetic modifications targeting the germline of *Anopheles* species (Meredith et al. 2013 ; Biedler et al. 2015 ; Macias et al. 2017; Terradas et al. 2021). Here, we utilize the *nanos* locus of *A. gambiae* for the integration of an IGD construct designed to express Cas9 protein under the control of *nanos* regulatory elements. The *nanos* IGD can trigger homing in the germline through a *nanos*-targeting gRNA expression cassette embedded within a synthetic intron. Blocking malaria transmission with the *nanos* IGD would be achieved through facilitating the spread of one or more nonautonomous antimalarial effectors (gRNA only) within the target population. To ensure safe use, the genetic modification in *nanos* IGD mosquitoes should not affect vector competence for malaria parasites or other relevant human pathogens. Here, we evaluate the feasibility and effectiveness of this gene drive and assess its potential for disease control.

## Materials and methods

### Generation of transgenic mosquitoes

The annotated DNA sequence file of the plasmid pD-Cas9-nanos is provided (https://osf.io/5h3bs/). Briefly, the intron containing the marker module and gRNA module is based on the first *A. gambiae gambicin 1* intron, which is 111 bp long. Three potential insertion sites for the two modules were tested with the *Drosophila* splice predictor, and the modules were placed between the bases ATA and GGA (Reese et al. 1997). The intron was inserted into the E2A autocleavage peptide between the bases TAC and GCC, and the last C was silently recoded to G, to match the endogenous flanking regions in *gambicin* exons 1 and exon 2. Two fragments were synthesized by GeneWiz Ltd., the first spanning the first part of E2A and the intron, as well as loxP and the rabbit globin terminator 3′ UTR to terminate GFP, and the second one spanning the second part of the intron and E2A. They were cloned into an intermediate plasmid to assemble the entire E2A containing the intron with the GFP module and the *A. gambiae* U6 promoter and scaffold for the gRNA as previously published (Hoermann et al. 2021). Approximately 800 bp of homology arms were amplified from *A. gambiae* Ifakara genomic DNA and incorporated at the 5′ and 3′ ends of the construct. The *nanos* gRNA was chosen using CRISPOR (Concordet and Haeussler 2018), checked for conservation with the Ag1000G database (Anopheles gambiae 1000 Genomes Consortium et al. 2017) and inserted via primers with overlaps during the final Gibson assembly. *A. gambiae* (Ifakara strain) eggs were injected with the donor plasmid pD-Cas9-nanos and the Cas9 helper plasmid p155 (Hammond et al. 2016) at a concentration of 200 ng/µL each, yielding 34 GFP-positive G_0_ individuals. Five G_1_ transgenics were obtained from a cage with 10 G_0_ females crossed to wild-type males. The insertion in G_1_ transgenics was confirmed by Sanger sequencing. The marker-free nanos^d^ line was then established by crossing nanos^D^ to a Cre recombinase-expressing line. This resulted in the excision of the GFP marker located between two loxP sites. The marker-negative individuals were intercrossed and confirmed to be nanos^d^ homozygous by pupal case PCR.

### Mosquito husbandry

*Anopheles gambiae* Ifakara strains were maintained at ∼27 °C and ∼70% humidity with a 12 h:12 h light:dark cycle and provided with 10% fructose *ad libitum*. All experiments were performed with cow blood (First Link Ltd.) except for parasite and virus infection experiments (human blood; Cambridge Bioscience).

### Assessment of gene drive

For the *nanos* drive autonomous homing assay, each replicate of heterozygous nanos^D^/nanos^d^ males or females was crossed to wild-types (pool sizes of 35 to 105 individuals), and their progeny were collected for subsequent genotyping. The inheritance of nanos^D^ was determined by the presence of GFP, whereas the inheritance of nanos^d^ was obtained by genotypic PCR with primers 663, 716, and 717. For nonautonomous homing of MM-CP, 45 to 50 of nanos^d^;MM-CP double-heterozygous males or females were crossed to wild-type mosquitoes, the progeny were collected and underwent genotypic PCR for nanos^d^ (using primers 663, 716, and 717) and for MM-CP (using primers 260, 531, and 532). Estimated means and 95% confidence intervals were calculated by a generalized linear mixed model, with a binomial (‘logit” link) error distribution fitted using the glmmTMB package (McGillycuddy et al. 2025). Replicates were included as a random effect, and parameters included drive carrying sex, the different drives (nanos^D^ and nanos^d^), and the sex of the Cas9 carrying grandparent. For the crosses including MM-CP, the sex of the gRNA-carrying grandparent was added as a parameter, and the drive options being MM-CP and nanos^d^.

### Amplicon sequencing

Genomic DNA was extracted with the DNeasy Blood & Tissue Kit (Qiagen) from approximately 400 L2/L3 larvae progeny from male or female heterozygotes nanos^d^ that were crossed to wild-type mosquitoes. PCRs were performed with Q5 High-Fidelity DNA Polymerase (NEB) using primers 717 and 737. Annealing temperature, extension time, and cycle number were set to 63 °C, 10 s, and 30 cycles, respectively. Amplicons were purified with the QIAquick PCR Purification Kit (Qiagen) and submitted to Amplicon-EZ NGS (GeneWiz Ltd.), and the data were analyzed with CRISPRESSO2 (Clement et al. 2019) and visualized with R (R Development Core Team).

### Pupation timing and adult survival assays

Three replicates of 100 to 105 homozygous nanos^d^ or wild-type larvae were maintained in trays under laboratory conditions. The number of days required to develop from the L1 larvae to pupae was recorded, and the sex ratio was determined by pupal sexing. For the adult survival assays, two replicates of 50 nanos^d^ or wild-type adults were maintained separately in male and female cages and provided with 10% fructose under laboratory conditions. The number of dead mosquitoes was recorded daily to monitor the life span of each population.

### RNAseq analysis

Ovaries from 2 to 4 d post-emergence, unmated homozygous nanos^d^ and wild-type females were dissected. Ovary samples were homogenized with a sterile pestle, and RNA extractions were performed using the Direct-zol RNA Microprep kit (Zymo Research), including the DNase treatment step. A total of 30 ovary pairs per line were collected for three biological replicates. The mRNA samples were purified using poly-T oligo-attached magnetic beads, and cDNA libraries were generated by using dUTP. The cDNA libraires were sequenced on a NovaSeq X Plus Illumina platform, generating 150 bp paired-end reads (NAR accession: PRJNA1327786). Sequencing reads were aligned to the *A. gambiae* PEST genome (AgamP4.13, GCA_000005575.2), supplemented with the nanos^d^ sequence reference, using HISAT2 v2.0.5 with the parameters –dta and –phred33 (Kim et al. 2015). Differential gene expression was analyzed using DESeq2 v1.20.0, and gene ontology (GO) enrichment analysis was conducted using g:Profiler, applying a *P*-value cutoff of 0.01.

### Parasite and virus infections

Wild-type or nanos^d^ adult mosquitoes were exposed to an infectious blood meal containing mature *P. falciparum* NF54 gametocytes (approximately 0.5% parasitemia in the infectious blood) using a streamlined standard membrane feeding assay or with *P. berghei* ANKA 2.34 by direct feeding on infected mice (2 to 3% parasitemia). Engorged mosquitoes were maintained at 27 °C or 21 °C for *P. falciparum* and *P. berghei* infections, respectively. For both *P. falciparum* and *P. berghei* infection experiments, 10% fructose was provided to the mosquitoes from 48 h post-blood meal to eliminate unfed individuals. O’nyong nyong virus Ahero strain (KX771232.1) was used for virus infection experiments. Three to four-day-old adult mosquitoes were provided a mixture of ONNV and human red blood cells to a final titer of 1 × 10^7^ PFU/mL. The infectious blood meal was conducted at 37 °C for no more than 30 min, and the mosquitoes were subsequently immobilized on ice to remove non-engorged individuals. The engorged mosquitoes were maintained at 27 °C and 80% relative humidity. At 7 d post-infection, the mosquitoes were immobilized on ice and their legs and wings were removed for the salivation assay. Mosquito saliva was collected by inserting proboscises into a P20 pipette tip filled with 5 µL of fetal bovine serum (FBS). After 30 min, the saliva-containing FBS was expelled into 45 µL of Dulbecco's Modified Eagle media and stored at −80 °C until analysis. After salivation, the mosquitoes’ abdomens and head-thoraxes were separated and stored individually for viral infection and dissemination assays, respectively.

### Population cage experiments

Male and female pupae of nanos^d^ and MM-CP (which carries a *zinc carboxypeptidase A1*-targeting gRNA (Hoermann et al. 2022)) mosquitoes were allowed to emerge in separate cages and mixed to mate as adults. For each replicate, 100 heterozygous nanos^d^, 100 heterozygous MM-CP mosquitoes for each sex were seeded at G_0_, totaling 400 individuals. For each generation of the cage trials, adults were blood fed at approximately six days post-emergence and allowed to oviposit into two egg bowls filled with water and lined with filter paper. The hatched larvae were transferred to trays with 105 larvae each and were maintained until the pupal stage to seed the next generation of the cage trial. For the four replicate cages that included nanos^d^ and the MM-CP transgene, larvae at L2/L3 stage were randomly selected from each generation and placed in a 96-well plate (92 larvae and 4 negative controls: three DNA extraction buffer and one water) and gDNA was extracted with the Phire Tissue Direct PCR Kit (Thermo Scientific). Multiplex PCRs were performed with primers 663, 716, and 717 for nanos^d^, and primers 260, 531, and 532 for MM-CP.

### Statistics, visualization, and reproducibility

Data analysis and visualization were performed with R. Code to reproduce plotting and statistical analysis performed in R is provided. No statistical method was used to predetermine sample size prior to experiments. No data were excluded from the analyses. Diagrams were generated with Inkscape.

## Results

### CRISPR-Cas9-mediated integration of an IDG construct at the *nanos* locus

**Fig. 1.**
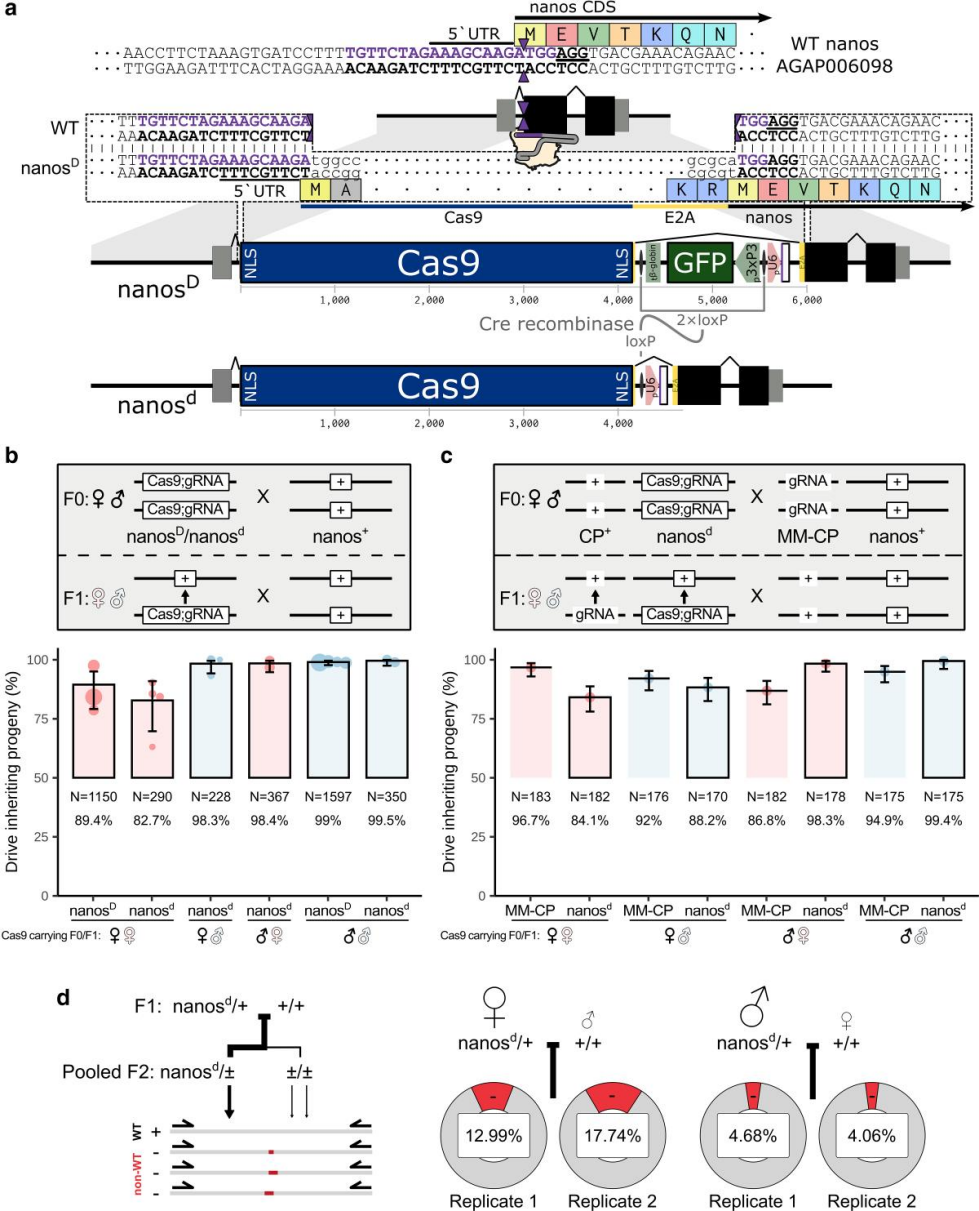
The *nanos* IGD. a) Schematic of the *nanos* IGD. The sequence of the *nanos* locus, gRNA target site (sequence in purple), PAM site (underlined), and CRISPR-Cas9 cutting site (purple triangles) are highlighted. To generate nanos^D^, the *nanos* IGD construct was integrated immediately upstream of the *nanos* CDS via homologous recombination. The marker-free nanos^d^ line was established by crossing nanos^D^ mosquitoes to a Cre recombinase line. b) Drive transmission from heterozygous male or female (colored gender symbols) nanos^D^ and nanos^d^ individuals. c) Drive transmission from a double heterozygous male or female nanos^d^;MM-CP individuals. Sex of the nanos^D^ or nanos^d^ grandparent is indicated with black gender symbols (contribution of MM-CP is from the opposite sex). Each point represents the mean from a pooled independent biological replicate, with the GLM estimated mean and the total number of offspring scored listed. Bars start at the expected inheritance rate and extend to the observed overall mean inheritance. d) The proportion of indels identified by amplicon sequencing analysis of the offspring of male and female nanos^d^ heterozygotes crossed to wild-type mosquitoes. The + symbol represents a wild-type allele; the − symbol represents a resistance allele; the ± symbol represents either.

An IGD construct was engineered for insertion into the *nanos* locus of *A. gambiae* (AGAP006098) via CRISPR-Cas9-mediated homologous recombination (Fig. 1a). The construct includes an NLS-Cas9-NLS coding sequence with an E2A skipping peptide facilitating in-frame translation with the endogenous *nanos* coding sequence, which is flanked by *nanos* homology arms. The E2A skipping peptide between the coding sequences of Cas9 and *nanos*, is intended to cause ribosome skipping *i.e.* the ribosome fails to form the Gly–Pro peptide bond, resulting in the separation of two proteins from a single open reading frame (Sharma et al. 2012 ; Daniels et al. 2014). A synthetic intron that contains a gRNA and green fluorescent protein (GFP) marker expression cassette was embedded within the E2A skipping peptide sequence. Upon embryonic microinjection, Cas9 provided by a helper plasmid and *nanos*-targeting gRNA transcribed from the donor plasmid mediate HDR at the target site. A gene drive strain carrying a GFP selection marker (nanos^D^) was generated through crossing the GFP-positive G_0_ individuals with wild-type mosquitoes.

IGDs are designed to express nucleases under the control of endogenous regulatory elements while preserving the normal function of the host gene, but we anticipated that this may be impacted by the presence of the GFP reporter cassette within the synthetic intron. A marker-free strain (nanos^d^) was established by outcrossing to a transgenic mosquito line expressing Cre recombinase to excise the selection marker flanked by loxP sites.

### Nanos^d^ shows a strong inheritance bias that is affected by maternal deposition

High inheritance rates were observed for both the nanos^D^ and markerless nanos^d^ drive lines which we assessed via fluorescent or PCR-based screening of F2 individuals, respectively (Fig. 1b). To evaluate maternal deposition, we performed the crosses such that the drive allele was contributed to the assessed heterozygotes by a grandparent of a specific sex (*e.g.* drive allele contribution by a male F0, to a female F1 is recorded as ♂♀). Crosses with the GFP marker (♀♀: 89.4%, ♂♂: 99.0%), and without (♀♀: 82.7%, ♂♂: 99.5%) were not statistically different in their inheritance rates (*P* = 0.63). However, the contribution of the markerless drive from female grandparents (♀♀: 82.7%, ♀♂: 98.3%) resulted in significantly lower inheritance rates than those with paternal (♂♀: 98.4%, ♂♂: 99.5%) nanos^d^ contribution (*P* = 0.04). In general, inheritance rates from female heterozygotes were lower than those from males (*P* = 0.03).

### Nanos^d^ can effectively bias the nonautonomous drive element MM-CP

We assessed the ability of nanos^d^ to simultaneously induce homing of a non-autonomous antimalarial effector (MM-CP) that resides in and carries a gRNA targeting the *zinc carboxypeptidase A1* (CP) gene (Hoermann et al. 2022). MM-CP expresses two antimalarial peptides in the midgut after blood meal ingestion and is a promising candidate effector to be disseminated using homing drive systems (Supplementary Fig. 1a; Hoermann et al. 2022). Male and female nanos^d^;MM-CP double-heterozygous F1 individuals were generated by crossing homozygous nanos^d^ and MM-CP mosquitoes. As in the previous crosses, the sex of the nanos^d^ carrying grandparent is listed as it alone carries Cas9, which the MM-CP grandparent lacks. Double-heterozygous F1 individuals were crossed with wild-type mosquitoes, leading to efficient inheritance rates of both the nanos^d^ (♀♀: 84.1%, ♀♂: 88.2%, ♂♀: 98.3, ♂♂: 99.4) and MM-CP (♀♀: 96.7%, ♀♂: 92.0%, ♂♀: 86.8, ♂♂: 94.9) elements (Fig. 1c). Overall inheritance rates did not significantly differ between the two loci (*P* = 0.07); however, inheritance was significantly higher in the crosses involving a male Cas9 grandparent (*P* < 0.01). Notably, the nanos^d^ inheritance rate was higher than MM-CP in the crosses with male nanos^d^ grandparents (♂♀/♂♂), while the MM-CP inheritance rate was higher in the crosses with female nanos^d^ grandparents (♀♀/♀♂). This would be consistent with the effect of maternal deposition being more substantial for nanos^d^, as both gRNA and Cas9 are present in the grandmother, while the MM-CP gRNA element was always contributed by the grandparent lacking Cas9. Crosses with a GFP-marked CP element and nanos^d^ showed similar inheritance rates to those with MM-CP (Fig. 1c).

### Analysis of resistance alleles generated by maternal nuclease deposition

To further investigate the impact of maternal deposition and the nature of the resistance alleles it generated, pools of nanos^d^-heterozygous males (N = 40 and 49) and females (N = 20 and 40) were crossed with wild-type mosquitoes, and the non-nanos^d^ alleles, expected to include both unmodified as well as mutant alleles resulting from a failure to induce HDR, were amplified and sequenced in approximately 400 L2/L3 larvae offspring. Insertions, deletions, and substitutions caused by non-homologous end joining (NHEJ) were identified within the *nanos* locus in 4.06 and 4.68% of reads from male heterozygous nanos^d^ parents, and in 12.99 and 17.74% of reads from female heterozygous nanos^d^ parents (Fig. 1d), indicating a surplus of resistance alleles which are generated as a result of maternal deposition. In line with this, the mutant alleles recovered from the male cross have low diversity, suggesting F1 germline mutations were inherited by multiple offspring while the mutations in the female cross were diverse, thus implying F2 mosaicism due to maternal deposition of Cas9 (Supplementary Fig. 2). These indels are expected to disrupt Cas9;gRNA binding, forming cleavage-resistant alleles against, potentially limiting the spread of the gene drive. Interestingly, while many of the observed mutations were predicted to abolish *nanos* expression, a significant set of alleles from maternal nanos^d^ crosses might have reconstituted the *nanos* start codon (Supplementary Fig. 2). The identified resistance alleles may not be fully functional, as they were detected in F2 larvae prior to selection through the pupal and adult stages. However, it is possible there may already be some level of active selection at the embryo stage, which could explain the high level of mutations that plausibly recreate functional alleles.

### Nanos^d^ reduces female fertility and mosquito survival

**Fig. 2.**
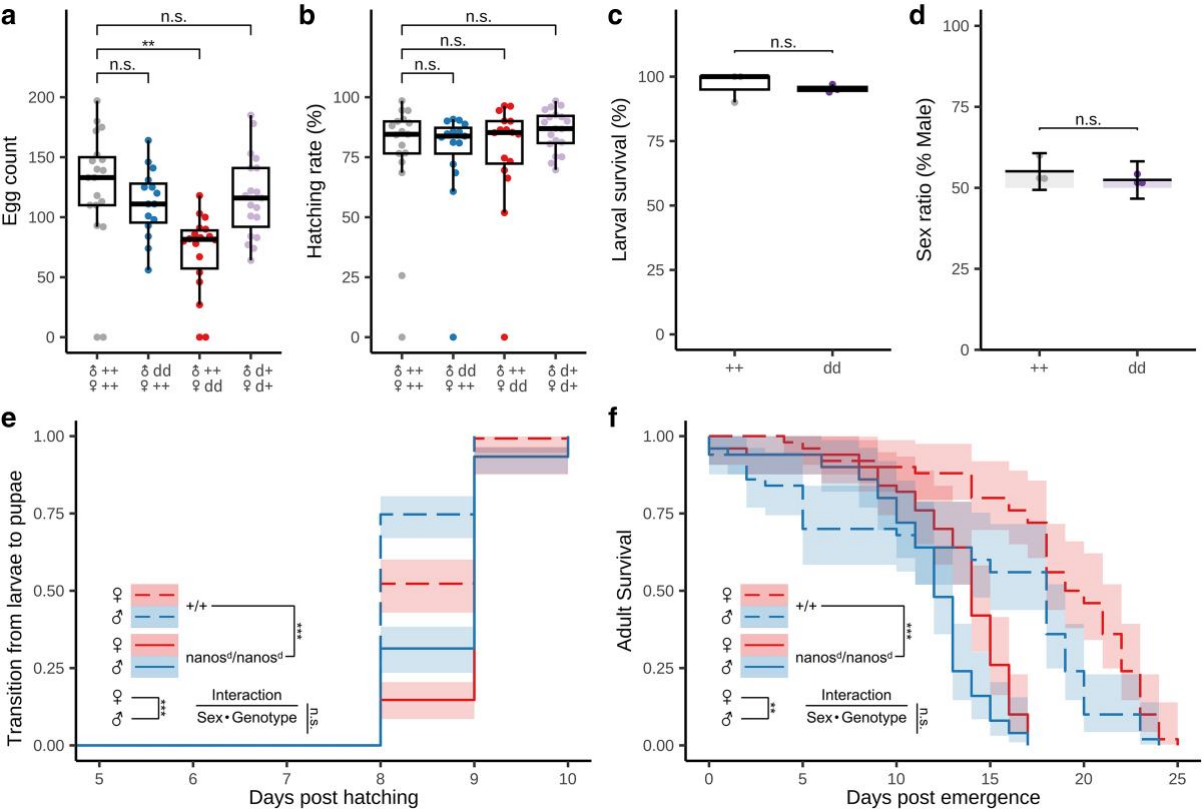
Fitness and life history traits of nanos^d^ mosquitoes. a) Fecundity and b) fertility of females with different nanos^d^ zygosity, or crossed to nanos^d^ carrying males. The heterozygous nanos^d^ mosquitoes in the fecundity and fertility tests were the progeny of male nanos^d^ crossed to female wild-type mosquitoes. Egg count significance levels were calculated using an unpaired two-tailed Student's *t*-test using a Bonferroni correction. c) Larval survival, d) pupal sex ratio, and e) development time from larval to pupal stages for homozygous nanos^d^ individuals. For b–d, significance levels were calculated using a binomial GLM with replicate as a random effect and Dunnett's multiple comparisons test. f) Averaged daily survival of homozygous male or female nanos^d^. The time series analysis in e and f was conducted using a mixed-effects Cox proportional hazards model. Shaded areas indicate the 95% pointwise confidence intervals. The ++ symbol represents wild-type mosquitoes; the d+ represents nanos^d^;wild-type heterozygous mosquitoes; the dd represents nanos^d^ homozygous mosquitoes.

Next, the life history traits of mosquitoes with the nanos^d^ gene drive were evaluated to identify potential unintended fitness costs. Fecundity was measured for different nanos^d^ zygosities by counting the number of eggs laid by individual inseminated females (Fig. 2a). No significant differences in egg counts were observed in crosses between nanos^d^ homozygous males and wild-type females, or between intercrossed nanos^d^ heterozygous males and females (male nanos^d^ grandparent). However, nanos^d^ homozygous females mated with wild-type males produced significantly fewer eggs compared to wild-type females, an effect that was similar in nanos^D^ mosquitoes (Supplementary Fig. 3). Aside from reduced fecundity, no significant differences were observed for the hatching rate (Fig. 2b), larval survival (Fig. 2c), or the sex ratio (Fig. 2d) between nanos^d^ and wild-type mosquitoes. Homozygous nanos^d^ mosquitoes did, however, exhibit an increased time to pupation (Fig. 2e) and a somewhat reduced adult lifespan (Fig. 2f).

### Nanos^d^ females show ovarian underdevelopment and reduced *nanos* expression

**Fig. 3.**
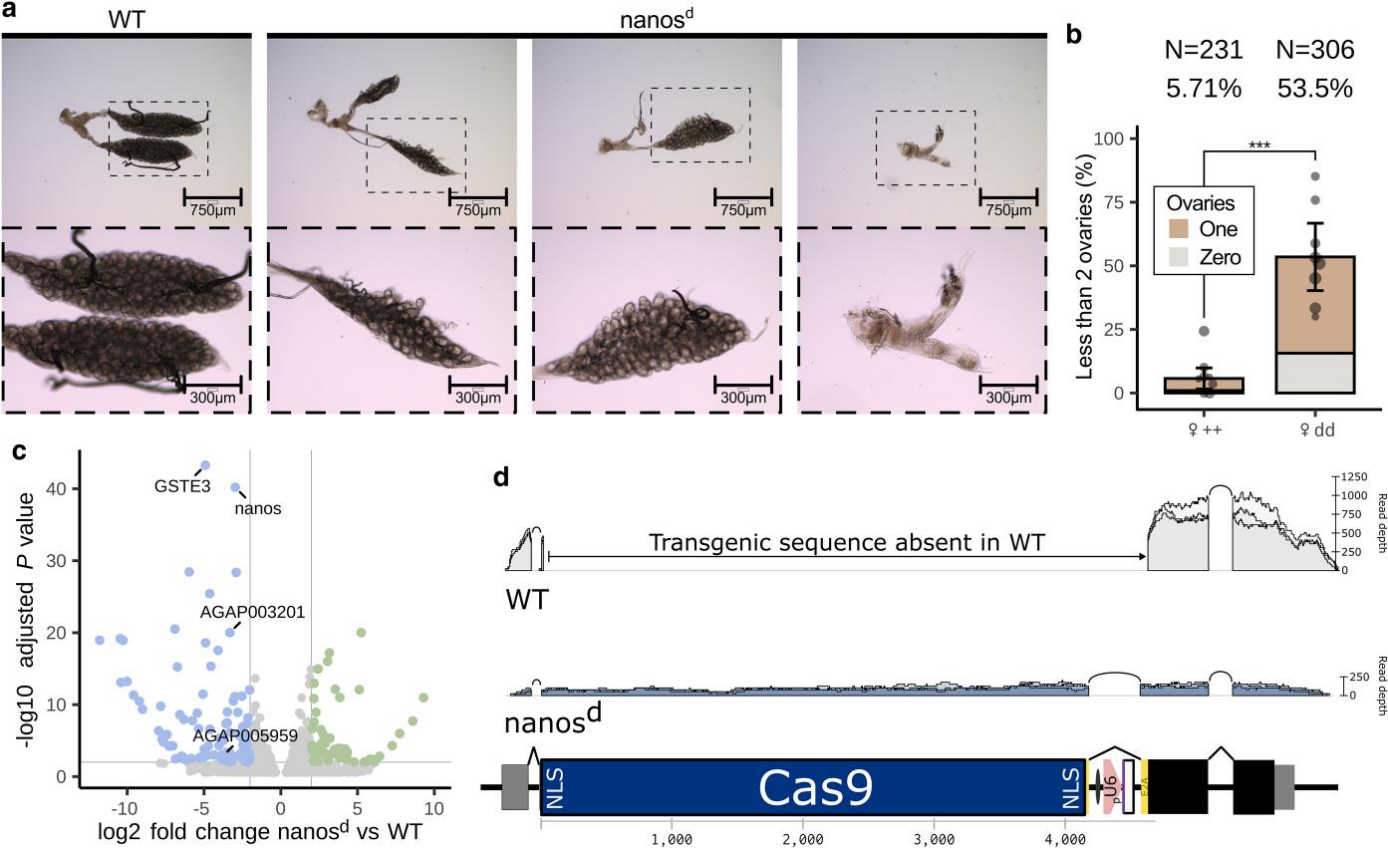
Development and gene expression profile of nanos^d^ mosquito ovaries. a) Ovarian morphology of wild-type and nanos^d^ mosquitoes, indicating severe developmental defects in nanos^d^ ovaries, which appear underdeveloped and more dispersed. b) Number of underdeveloped ovaries in nanos^d^ and wild-type mosquitoes. Mean estimates and significance levels were calculated using a multinomial GLM with replicate as a random effect. The symbol ++ represents wild-type mosquitoes; dd represents nanos^d^ homozygous mosquitoes. c) Volcano plot of differentially expressed genes in nanos^d^ and wild-type ovaries of 2 to 4-day-old mosquitoes, notable transcriptomic changes were highlighted, including genes involved in germline development. Differentially expressed genes between nanos^d^ and wild-type mosquitoes, identified based on a significance threshold of *P* ≤ 0.01 and a log₂-fold change ≥ 2, are colored. d) Reads mapped to the *nanos* locus and the nanos^d^ construct, demonstrating Cas9 expression and confirming correct Cas9 gene splicing in the ovaries of nanos^d^ mosquitoes. The total number of reads mapped to the genome for the two groups was similar (WT: 4.2 × 10^7^; nanos^d^: 4.1 × 10^7^).

The reduced fecundity phenotype was further investigated by dissecting and examining mosquito ovaries (Fig. 3a). Results showed that 53.5% of sugar-fed nanos^d^ mosquitoes had underdeveloped ovaries, whereas only 5.71% of wild-type mosquitoes exhibited the same phenotype (Fig. 3b). In addition to the differences in ovary development, the spacing between individual ovarioles in developed nanos^d^ ovaries appeared more dispersed compared to those in fully developed ovaries from wild-type mosquitoes. We next performed transcriptome analysis on the ovaries from nanos^d^ and wild-type females (Fig. 3c). Gene expression profiling revealed a significant reduction in *nanos* expression levels, with only one other gene, glutathione S-transferase *GSTe3* (AGAP009197), known to be upregulated in DDT-resistant strains (Ding et al. 2003), reaching a similar level of significance (Fig. 3c). Some female fecundity-related genes were significantly downregulated in the ovaries of nanos^d^ mosquitoes and may be linked to the reduced fecundity observed in this study. Notably, the maternally-deposited *Drosophila Tao-1* gene (AGAP003201), which is involved in cell migration during embryogenesis in *Drosophila* (Pflanz et al. 2015), and a fertility related gene, *yellow-g2* (AGAP005959), were downregulated in the ovaries of nanos^d^ mosquitoes. In *Aedes albopictus*, ovary-expressed *yellow-g2* encodes a dopachrome conversion enzyme essential for cuticle melanization, which is required for egg desiccation resistance and pigmentation (Noh et al. 2020). The RNA sequencing data also allowed us to confirm that the additional intron we introduced as part of the nanos^d^ construct did not lead to unintended splicing products (Fig. 3d).

### *Nanos^d^* does not alter vector competence for *Plasmodium* and O’nyong nyong virus

**Fig. 4.**
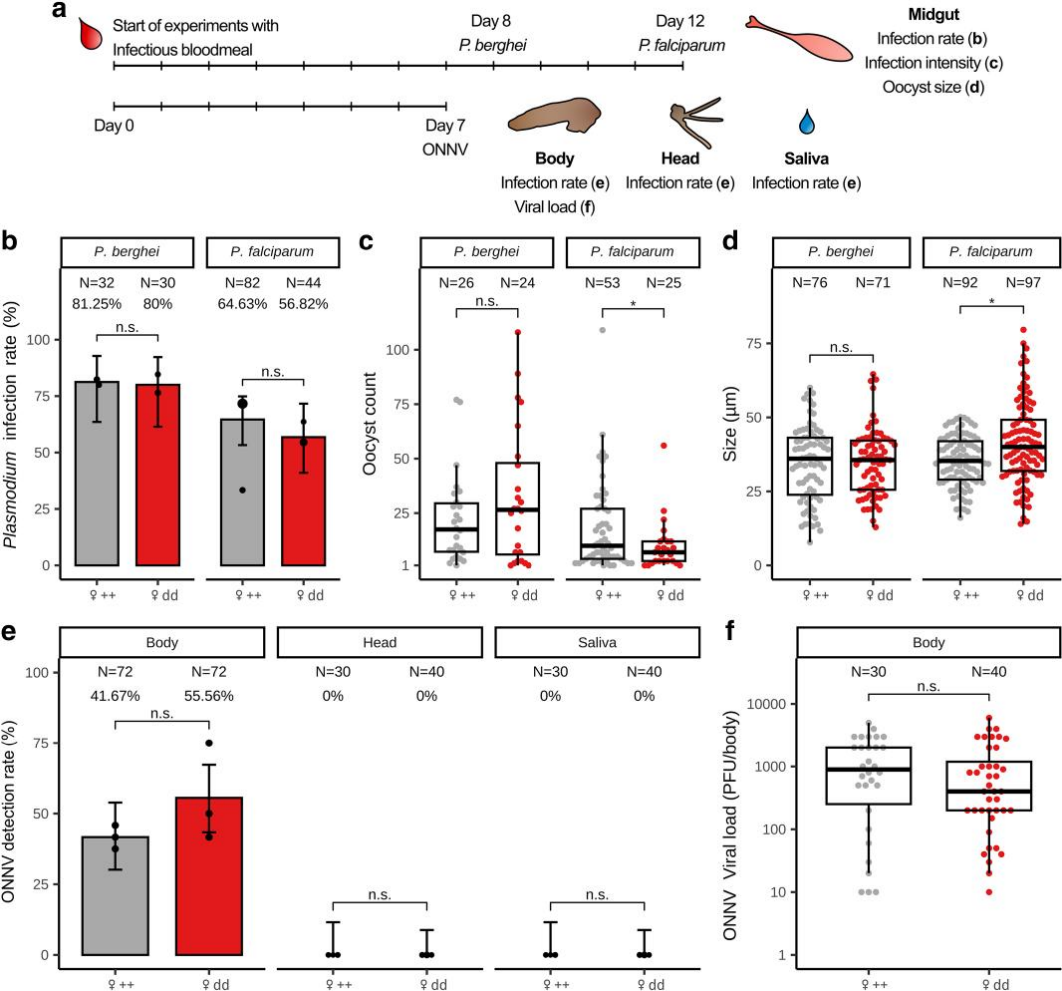
Vector competence of nanos^d^ mosquitoes. a) Outline for *Plasmodium* spp. (upper) and ONNV infection experiments (bottom). b) *P. falciparum* and *P. berghei* oocyst infection rate, c) infection intensity, d) and oocyst size in the midgut of wild-type and nanos^d^ mosquitoes at 8 or 12 d post-blood feeding, respectively. Pooled results of two replicates are presented. The median oocyst diameter is indicated by the black bar. e) ONNV detection rate in mosquito bodies, heads, and saliva at 7 d post-blood feeding. f) Viral load comparison between nanos^d^ and wild-type midguts. Means and significance levels for b and e were calculated using a binomial GLM with replicate as a random effect. Significance levels for c, d, and f were calculated using an unpaired two-tailed Student's *t*-test using a Bonferroni correction. The symbol ++ represents wild-type mosquitoes; dd represents nanos^d^ homozygous mosquitoes.

The nanos^d^ gene drive is neither intended nor expected to affect vector competence, as it is designed to be combined with, and to propagate, separate effector traits responsible for the antimalarial effect. We nonetheless evaluated the susceptibility to infection of nanos^d^ mosquitoes in case of unintended effects of this modification (Fig. 4a). The results showed that female nanos^d^ mosquitoes do not exhibit an increased infection rate (prevalence) for the rodent malaria parasite *Plasmodium berghei,* or for the human parasite *Plasmodium falciparum* (Fig. 4b). In fact, *P. falciparum* oocyst intensities were slightly lower in nanos^d^ mosquitoes compared to wild-type controls (Fig. 4c), accompanied by slightly larger oocyst diameters, likely due to reduced competition for resources among parasites (Fig. 4d). We also assessed vector competence for O’nyong nyong virus (ONNV). Again, nanos^d^ mosquitoes exhibited no increase in infection rates (Fig. 4e) or viral replication (Fig. 4f). The absence of detectable viral particles in the heads and saliva of infected mosquitoes suggests a low risk of ONNV dissemination and transmission, indicating that the genetic modification does not enhance virus replication.

### Nanos^d^ and MM-CP propagate efficiently in cage populations

**Fig. 5.**
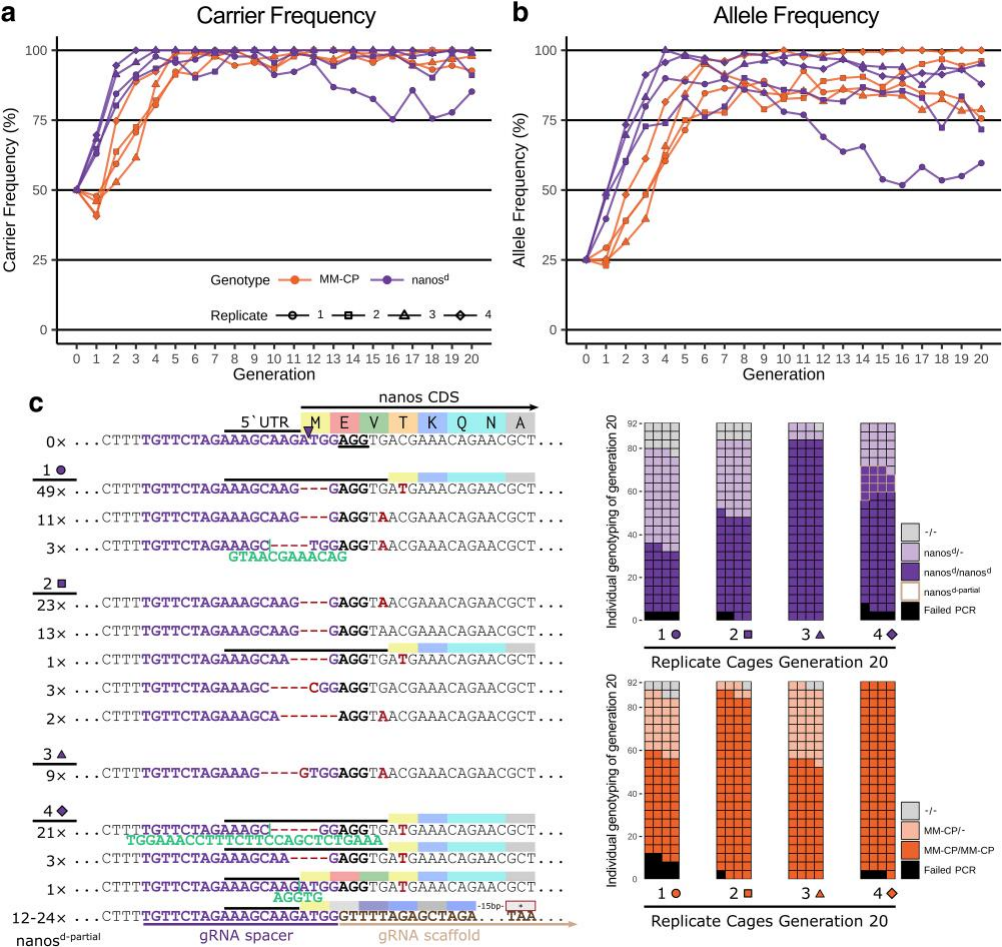
Cage population invasion experiments. a) Carrier and b) allele frequency of nanos^d^ (purple) and MM-CP (orange), in four independent trials over 20 generations. Populations were seeded with 50% nanos^d^ and 50% MM-CP heterozygotes. c) Nondrive alleles identified by sequencing the *nanos* locus at generation 20. Reference wild-type sequence for *nanos* is shown, with the PAM underlined and the gRNA cut-site indicated by an arrow. The number of times each allele was encountered is indicated, with deleted nucleotides shown as –, insertions shown in green, and substitutions shown in red. Amino acid translations are shown starting at the first exonic ATG. The waffle plots represent genotype composition of nanos^d^ (upper) and MM-CP (bottom) in each cage at generation 20.

We next performed cage trials to test whether the inheritance bias and fitness effects observed in single-generation crosses translate into multigenerational studies. Four cages were seeded with 400 individuals, consisting of 200 nanos^d^ heterozygotes and 200 MM-CP heterozygotes at a 1:1 sex ratio. This corresponds to an initial allele frequency of each transgene of 25% (50% carrier frequency) for nanos^d^ or MM-CP. Transgene carrier (Fig. 5a and Supplementary Fig. 4) and allele (Fig. 5b and Supplementary Fig. 5) frequencies were monitored over 20 discrete generations using multiplexed PCR genotyping, as neither line carries a fluorescent marker. Both transgenes spread rapidly, reaching a plateau after 5–10 generations, with nearly all individuals carrying at least one copy of each transgene. As expected, MM-CP trailed behind nanos^d^ in frequency, as its biased inheritance requires the presence of nanos^d^ in the same individual, which could not occur in the initial release generation. In the first replicate, the nanos^d^ frequency began to decline after generation 7, suggesting the emergence of drive-resistant alleles that are fitter than the nanos^d^ drive allele. However, the subsequent stabilization of the nanos^d^ frequency suggests that there is less selection against the drive when it is not in homozygosis.

### Identification of nanos^d^-resistant alleles in cage populations

To characterize potentially drive-resistant alleles in these cage populations, the DNA of individuals that lacked nanos^d^ was sequenced. The results revealed a range of alleles invariably disrupting the start codon of the *nanos* gene (Fig. 5c), many of which were also prominent in our sequencing of pooled crosses (Supplementary Fig. 2). However, these mutations were frequently found in combination with a downstream C to T substitution that introduces an alternative start codon (Thr4Met; Fig. 5c; Supplementary Fig. 6c), likely resulting in production of *nanos* with a three-amino acid N-terminal truncation. These variants are particularly dominant in replicate 1 and may preserve *nanos* function and thus confer resistance to the drive without imposing a major fitness cost.

While performing additional PCRs for sequencing generation 20, we unexpectedly identified partial drive alleles (nanos^d-partial^) in replicate 4, which lost most of the 5′ region of the drive. These alleles were classified as drive alleles as they still retain the internal primer binding sites we used for genotyping. We noted that the deleted sequence stretches between a 20-nucleotide repeat formed by the gRNA target site (missing the PAM) flanking the 5′ of the intact drive, and the gRNA spacer in the synthetic intron (Supplementary Fig. 6a). The deletion could have been mediated by instability caused by the flanking direct repeats, which has also been observed in multiplexed gene drives with repeated use of an identical gRNA scaffold or promoter (Green et al. 2023). However, the repeated sequence is notable as it also exactly aligns with one of the ends of the cut wild-type allele, suggesting the partial allele was formed through homing that was erroneously initiated at this internal stretch of homology (Hammond et al. 2016; Verkuijl et al. 2022*)*. This nanos^d-partial^ allele was identified exclusively in replicate four, where 12 out of 87 samples carried the allele, corresponding to a carrier frequency of 14% at generation 20 (Fig. 5c). We performed additional sequencing of the first generation of replicate four and did not identify the partial drive allele (Supplementary Fig. 6b), suggesting it was created due to drive activity during the cage trial. In sequencing the first generation and in our wild-type RNAseq samples, we also noted a high frequency of wild-type alleles containing the Thr4Met variant, indicating it is a preexisting variant, not a separate adaptive mutation.

In contrast to the seemingly functional nanos^d^ alleles, we found a lower diversity of resistance alleles for CP, which were primarily large deletions (Supplementary Fig. 6c). Given that MM-CP frequencies remained high even when nanos^d^ frequencies declined, these mutations are unlikely to confer a selective advantage, consistent with them being non-functional. This contrast with nanos^d^ is notable, as the cut-site at the CP allele is slightly upstream of the start codon in the 5′ UTR, which may be expected to be more prone to generating functional resistance alleles.

## Discussion

Significant efforts are required to overcome a set of interwoven technical, social, and regulatory hurdles for field testing and the eventual deployment of gene drives. Gene drive systems designed with these aspects in mind might be more readily adopted. The IGD paradigm offers to closely recapitulate endogenous germline expression and minimize the genetic footprint of an autonomous gene drive modification. Effector modifications targeting the parasite, such as the MM-CP effector, must be active in non-germline tissues, and as such must be housed at distinct loci with appropriate expression patterns. However, separating the effector and inheritance bias modules comes with the benefit of phased testing approaches, which can provide a more gradual path to approval. The linkage of the antimalarial or drive components to an endogenous gene may also increase transgene integrity by selection against loss-of-function mutations that also affect the host gene (*e.g.* frameshift mutations). The nanos^d^ gene drive described here is a promising integral drive element that, with further optimization, can support the effective spread of nonautonomous antimalaria effectors.

IGDs in *A. gambiae* and *A. stephensi* utilizing the *zero-population growth* (*zpg*) locus for Cas9 expression have been previously reported, demonstrating efficient homing drive performance (Ellis et al. 2022). The fitness costs in *A. stephensi* have not yet been described (Gonzalez et al. 2025; Larrosa-Godall et al. 2025). However, in *A. gambiae,* these *zpg* IGDs suffered from a dominant loss of fecundity in female carriers, which limited their use for population modification. This suggested that the *zpg* host gene does not easily tolerate modification and that its function was severely perturbed following the insertion of a Cas9 coding sequence. In this study, we have utilized the *nanos* gene of *A. gambiae* to develop an autonomous IGD element. The nanos^d^ gene drive element makes use of the regulatory regime of the *nanos* host locus and supports high levels of inheritance bias. Despite the removal of the intronic fluorescent marker cassette, some fitness parameters, including fecundity, pupal developmental time, and adult lifespan, were affected in nanos^d^ individuals. The reduced *nanos* expression level could be explained by the existence of a potential enhancer downstream of the *nanos* 5′ UTR, where the large nanos^d^ modification resides. A similar reduction was observed in the AgNosCd-1 gene drive, in which a 1.6 kb *nanos* promoter fragment was used to drive Cas9 at the *cardinal* locus and which showed a marked reduction in expression when compared to endogenous *nanos* levels (Terradas et al. 2021). While further optimization is possible, nanos^d^ nevertheless represents a notable improvement over previous attempts at the *zpg* locus, and unlike these earlier designs in *A. gambiae*, the fitness profile of nanos^d^ would allow it to fix itself within a population.

We noted a decrease in inheritance bias from nanos^d^ heterozygotes that were generated through crosses that allowed for maternal deposition of Cas9. Maternal deposition of Cas9, or Cas9;gRNA, into the zygote can lead to cutting at stages when homing is not favored, and has been commonly observed for canonical *Anopheles nanos* drives (Hammond et al. 2021; Carballar-Lejarazú et al. 2022, 2023). Deposition can cause stochastic mosaic mutation patterns in germline and nongermline cell lineages alike. Crosses with male nanos^d^ and female MM-CP grandparents showed a lower MM-CP inheritance rate compared to nanos^d^. In contrast, crosses with female nanos^d^ and male MM-CP grandparents exhibited a lower nanos^d^ inheritance rate compared to MM-CP. These results suggest that the baseline inheritance rate of nanos^d^ is higher, but maternal deposition reduces nanos^d^ inheritance more than that of MM-CP. This would be consistent with simultaneous maternal deposition of both Cas9 and gRNA, affecting inheritance rates more negatively than deposition of Cas9 alone.

The IGD design requires the gene drive element to flank the coding sequence of the endogenous gene. This positioning causes the selection of viable gRNAs near the insertion site to often overlap with noncoding sequences of the target gene. When we sequenced the offspring of drive heterozygotes, many of the indels identified resulted in frameshift mutations that are predicted to disrupt correct protein translation. However, due to the cut-site overlapping the start codon of *nanos*, a range of indels that extended into the 5′UTR recreated an alternative start codon, possibly allowing for continued expression of functional *nanos*. Notably, in the cage trial, we identified separate mutations that restored the *nanos* reading frame through an alternative downstream methionine codon, present as a preexisting allele in our wild-type colony. A similar observation was reported for a homing drive targeting the *hairy* gene of *D. melanogaster*, where a downstream methionine codon restored protein function, subsequently leading to the formation of a functional resistance allele (Hou et al. 2024).

Functional mutations would plausibly explain why some cage experiments saw a decline in the nanos^d^ allele frequency. This accumulation may affect the persistence of nanos^d^ but need not impact the spread of the antiparasitic payload construct if a sufficiently high carrier frequency of Cas9 is reached within the population. Maintenance of a Cas9 source at an intermediate frequency could allow for the release and propagation of secondary nonautonomous effector traits into the population. Nonetheless, deposition of Cas9 by *nanos* is expected to also negatively affect the gRNA-expressing effector and should therefore be minimized. Overall, we describe an IGD with moderate fitness costs that can disseminate as an autonomous gene drive and propagate a non-autonomous antiparasitic payload construct. The nuclease profile of this strain can be further optimized by shifting the target site and decreasing the persistence of Cas9 into the embryo by reducing the protein stability. Designing gRNA sequences that target essential motifs further removed from the start codon is expected to mitigate the accumulation of the type of resistance alleles we observed. Moreover, employing multiple gRNAs targeting different sites within the locus could further reduce the likelihood of functional resistance allele formation.

We also identified a gene drive deletion allele. This was likely generated as a consequence of one of the DNA strands of the recipient chromosome, initiating DNA repair with the short homologous gRNA spacer sequence located within the drive construct, instead of the extended homologous sequence flanking the drive. Inversion of the gRNA cassette may reduce the likelihood of such events. It is worth noting that our multiplexed PCR-based genotyping makes it more likely to detect such deletion events than fluorescence-based screening, as, in addition to measuring presence/absence, failure to amplify either the drive or resistance alleles can indicate a more complex genotype. We have therefore also reported the frequency of these failures for the cage trial genotyping we performed, which remained relatively consistent between replicates and generations.

In *D. melanogaster*, the *nanos* gene is expressed in the early germarium, which maintains transcriptional quiescence and acts as an important determinant of posterior patterning during oogenesis (Wang et al. 1994; Hayashi et al. 2004). Tiny or agametic ovaries are commonly observed in fly mutants when *nanos* is unable to carry out its role in germline stem cell maintenance (Verrotti and Wharton 2000; Wang and Lin 2004). The *nanos* gene itself was one of the most significantly downregulated genes in nanos^d^ ovaries and thus explains the underdevelopment phenotype and fertility effects we observed. While mRNA transcription rate and stability may have been affected by the insertion, we cannot exclude that the insertion affects *nanos* in different ways, such as delayed translation, or loss of protein functionality due to insufficient separation at the 2A linker.

Notably, female nanos^d^ heterozygotes did not display fertility defects. In the tissues where *nanos* is expressed, so is Cas9; if homing occurs rapidly, one should expect similar impacts in heterozygotes and homozygotes. Moreover, in heterozygotes, the two *nanos* alleles will be engaged in DNA repair processes that could prevent expression at either allele. The unaffected fertility of heterozygotes could be due to the accumulation of wild-type *nanos* transcripts before the homing process is initiated. This would most likely be due to delayed translation, but could also be explained by the initial absence of gRNA expression when Cas9 is first translated. Additionally, a grandparental effect might contribute. In our assay, homozygotes for nanos^d^ would have received reduced maternal deposition of *nanos* transcripts from their nanos^d^ homozygous mothers, while heterozygotes from wild-type mothers would have received normal levels, potentially diminishing the effect during early developmental processes.

Another significant fitness impact was observed in adult lifespan, with both male and female nanos^d^ homozygous mosquitoes exhibiting reduced survival rates compared to wild-type. In general, *Anopheles* mosquitoes require 10–14 d to transmit sporozoites after ingesting human *Plasmodium* gametocytes (Ohm et al. 2018) and approximately 7 d for ONNV transmission (Mutsaers et al. 2023). The reduced lifespan of females, if attributable to the genetic modification itself, could be a desirable trait for reducing the risk of pathogen transmission. However, the underlying causes of the shortened lifespan in both sexes remain unclear. Given the low genetic diversity of transgenic strains, inbreeding could be an explanation for some of the somatic phenotypes observed. Although Cas9 expression may place a general metabolic load on cells, it is expected to be restricted to the germline at specific developmental stages and would be expected to have minimal impact on overall mosquito lifespan. The ubiquitous expression of the gRNA could also account for some systemic fitness effects. The reduction of *nanos* transcripts observed in the ovaries and its effect is expected to be confined to the reproductive system of mosquitoes and would not be expected to alter immune responses against pathogen infection. By contrast, the ubiquitous gRNA expression could trigger more systemic changes to immunity. We found that nanos^d^ mosquitoes showed no increased vector competence for *Plasmodium* or ONNV, although, as mentioned, their reduced adult lifespan would limit the chance for pathogen transmission and prevent the mosquito from becoming transmissible.

In conclusion, we present an IGD that can efficiently introduce itself and an antiparasitic effector into targeted *A. gambiae* populations. The drive element shows no enhanced vector competence for the most significant pathogens that *A. gambiae* transmits, and the shortened lifespan of nanos^d^ mosquitoes may further aid in reducing transmission. The observed fertility impact in nanos^d^ mosquitoes driving the selection of resistance alleles within the target population suggests directions for further improvements. Further studies are required to test this gene drive under more realistic environmental conditions and in combination with other antimalarial effectors.

## Supplementary Material

jkaf246_Supplementary_Data

## Data Availability

The supplementary figures underlying this article are available in the online Supplementary material. All code, raw data, and construct map are available in the OSF repository (https://osf.io/5h3bs/DOI: 10.17605/OSF.IO/5H3BS). Supplemental material available at G3 online.
