# Supplementary material for: The nanosd integral gene drive enables population modification of the malaria vector Anopheles gambiae
Source: G3 (Bethesda). 2025 Oct 29;16(8):jkaf246. doi: 10.1093/g3journal/jkaf246 (PMC13439923; doi:10.1093/g3journal/jkaf246)
Supplement: Supplementary file 1 [file jkaf246_supplementary_data.docx]

 **Figure S1. Targeted modifications at the CP locus and homing rates in *nanos*-IGD mosquitoes.** Schematic representation of two transgenic integrations at the CP locus and the inheritance rate of nanos^d^ mosquitoes that crossed to CP-KO mosquitoes. (a) The MM-CP construct incorporates magainin-2 and melittin under the regulation of CP promoter. Importantly, there is a gRNA:CP expression cassette embedded within the synthetic intron that separates Melittin. (b) The CP-KO construct includes a GFP marker, disrupting CP expression. The gRNA:CP targets a different sequence than the MM-CP in the CP-KO line. (c) Inheritance rates of nanos^d^ and CP-KO mosquitoes, measured as the percentage of progeny inheriting nanos^d^ and CP-KO. + represents wild-type alleles.

**Figure S2. Top 25 most abundant nanos^d^ resistance alleles found from male and female crosses.** Mutant alleles that recreate an ATG start codon are highlighted. Blue and green bars represent the percentage of each genotype from the two male crosses, whereas red and orange bars represent the two female crosses. The + symbol represents wild-type alleles; the - symbol represents resistance allele

**Figure S3. Fecundity and fertility of homozygous nanos integral gene drive mosquitoes with and without the intronic GFP cassette.** The number of eggs **(a)** and hatching rate **(b)** were recorded for intercrossed nanos^D^ or nanos^d^ females. ++ represents wild-type mosquitoes, DD represents homozygous nanos^D^; dd represents homozygous nanos^d^. Egg count significance levels were calculated using an unpaired two-tailed Student’s *t*-test using Bonferroni correction. Hatching rate means and significance levels were calculated using a binomial GLM with replicate as a random effect.

**Figure S4. CP allele counts in the population invasion experiments.** Composition of CP alleles across 20 generations in the four cage populations, each unit represents an individual mosquito. The cohorts used for seeding the next generation and for genotyping represented two independent random selections of mosquitoes. The ± symbol represents wild-type or resistance alleles, as the multiplexed genotyping PCR cannot distinguish between wild-type and resistance alleles.

**Figure S5. Nanos allele counts in the population invasion experiments.** Composition of nanos alleles across 20 generations in the four cage populations, each unit represents an individual mosquito. The cohorts used for seeding the next generation and for genotyping represented two independent random selections of mosquitoes. Note that the partial drive allele identified by sequencing of cage 4 generation 20 cannot be distinguished from the full drive by the genotyping PCR performed here. It is likely that some of the drive counts for generations 2-19 of cage 4 include the partial drive allele (it was confirmed to be absent in generation 1). The ± symbol represents non-drive alleles, as the multiplexed genotyping PCR cannot distinguish between wild-type and resistance alleles.

**Figure S6. Partial drive allele structure and sequencing of nanos R4 G1 and CP R1-4 G20. (a)** Schematic representation of the nanos^d^ allele and partial drive allele identified in the 20^th^ generation of the replicate 4 cage trial population. The extent of the homology of the gRNA expression cassette spacer sequence with the cut wild-type *nanos* locus is shown. The partial drive allele likely results in aberrant translation starting and terminating in the gRNA scaffold. **(b)** Alleles identified by sequencing of the *nanos* locus in the first generation of the 4^th^ cage trial replicate. No partial drive alleles were identified, suggesting this was not preexisting in our nanos^d^ colony and arose due to drive activity in this replicate of the cage trial. A high frequency of individuals was identified with a Methionine instead of a Threonine amino acid at the 4^th^ position, which may serve as an alternative start codon in some resistance alleles. **(c)** Alleles identified by sequencing of the CP locus in the 20^th^ generation of the cage trial replicates. All individuals genotyped in replicate 4 were homozygous for the MM-CP allele.
